# Hepatoprotective Potency of Chrysophanol 8-*O*-Glucoside from *Rheum palmatum* L. against Hepatic Fibrosis via Regulation of the STAT3 Signaling Pathway

**DOI:** 10.3390/ijms21239044

**Published:** 2020-11-27

**Authors:** Yong Joo Park, Kwang Ho Lee, Mi Seon Jeon, Yong Hoon Lee, Yoon Joo Ko, Changhyun Pang, Bonglee Kim, Kyu Hyuck Chung, Ki Hyun Kim

**Affiliations:** 1 School of Pharmacy, Sungkyunkwan University, Suwon 16419, Korea; pyj084@msn.com (Y.J.P.); sholaly@naver.com (K.H.L.); jmsun123@naver.com (M.S.J.); yonghoon2090@gmail.com (Y.H.L.); 2 Laboratory of Nuclear Magnetic Resonance, National Center for Inter-University Research Facilities (NCIRF), Seoul National University, Gwanak-gu, Seoul 08826, Korea; yjko@snu.ac.kr; 3 School of Chemical Engineering, Sungkyunkwan University, Suwon 16419, Korea; chpang@skku.edu; 4 Department of Pathology, College of Korean Medicine, Kyung Hee University, Seoul 02453, Korea; bongleekim@khu.ac.kr

**Keywords:** *Rheum palmatum*, chrysophanol 8-*O*-glucoside, hepatic stellate cells, hepatic fibrosis, STAT3

## Abstract

Rhubarb is a well-known herb worldwide and includes approximately 60 species of the *Rheum* genus. One of the representative plants is *Rheum palmatum*, which is prescribed as official rhubarb due to its pharmacological potential in the Korean and Chinese pharmacopoeia. In our bioactive screening, we found out that the EtOH extract of *R. palmatum* inhibited hepatic stellate cell (HSC) activation by transforming growth factor β1 (TGF-β1). Chemical investigation of the EtOH extract led to the isolation of chrysophanol 8-*O*-glucoside, which was determined by structural analysis using NMR spectroscopic techniques and electrospray ionization mass spectrometry (ESIMS). To elucidate the effects of chrysophanol 8-*O*-glucoside on HSC activation, activated LX-2 cells were treated for 48 h with chrysophanol 8-*O*-glucoside, and α-SMA and collagen, HSC activation markers, were measured by comparative quantitative real-time PCR (qPCR) and western blotting analysis. Chrysophanol 8-*O*-glucoside significantly inhibited the protein and mRNA expression of α-SMA and collagen compared with that in TGF-β1-treated LX-2 cells. Next, the expression of phosphorylated SMAD2 (p-SMAD2) and p-STAT3 was measured and the translocation of p-STAT3 to the nucleus was analyzed by western blotting analysis. The expression of p-SMAD2 and p-STAT3 showed that chrysophanol 8-*O*-glucoside strongly downregulated STAT3 phosphorylation by inhibiting the nuclear translocation of p-STAT3, which is an important mechanism in HSC activation. Moreover, chrysophanol 8-*O*-glucoside suppressed the expression of p-p38, not that of p-JNK or p-Erk, which can activate STAT3 phosphorylation and inhibit MMP2 expression, the downstream target of STAT3 signaling. These findings provided experimental evidence concerning the hepatoprotective effects of chrysophanol 8-*O*-glucoside against liver damage and revealed the molecular basis underlying its anti-fibrotic effects through the blocking of HSC activation.

## 1. Introduction

Hepatic fibrosis is a chronic liver disease characterized by excessive accumulation of extracellular matrix (ECM). ECM is a complex and dynamic component that can provide essential physical scaffolding to cells, such as structural support and elasticity, in all tissues and organs [1]. However, repetitive and chronic liver injuries promote ECM deposition as well as changes in matrix stiffness and flexibility, resulting in hepatic fibrosis [2]. Aberrant deposition of ECMs, such as collagens and fibronectin, increases rapidly during tissue injury and regeneration, and it is frequently observed in the space of Disse [3,4].

Hepatic stellate cells (HSCs), precursors of liver myofibroblasts, are resident mesenchymal cells and considered key cells in liver fibrogenesis. Quiescent HSCs are present in the sub-endothelial space of Disse, and they have resident fibroblasts and pericyte features containing cytoplasmic lipid droplets [5,6]. Chronic damage in parenchymal epithelial cells, such as hepatocytes, initiate inflammation and promote immune cell activation by releasing inflammatory mediators and damage-associated molecular patterns [7,8,9]. Cytokines, especially TGF-β1 from macrophages, are well-known fibrogenic agents that can activate HSCs [10]. Activated HSCs express α-smooth muscle actin (α-SMA) and release excessive ECMs, including collagens and cellular fibronectin, which are characteristic in progressive hepatic fibrosis [6,11,12]. Although HSCs constitute only 5–8% of the of the total number of liver cells, cell fate tracing in rodent-based studies show that appropriately 90% of myofibroblasts are derived from HSCs under treatment with fibrosis-inducing chemicals [13]. 

As HSCs are potential therapeutic targets in hepatic fibrosis pathogenesis, greater advances have been made in understanding the molecular mechanisms of HSC activation, and the inhibition and reversion of activated HSCs have been proposed as potential therapeutic strategies for hepatic fibrosis [14,15,16]. TGF-β1, a well-known pivotal cytokine in the development of hepatic fibrosis, clearly contributes to HSC activation and activates the signal transducer and activator of transcription 3 (STAT3) signaling [17,18]. The STAT3 is a transcription factor associated with the proliferation and activation of HSCs, and thus it can contribute to hepatic fibrosis [19,20]. In response to cytokines and growth factors after liver injury, the STAT3 is phosphorylated by Janus tyrosine kinases, dimerized, and translocated into the nucleus to transcript its target genes [21,22]. 

Rhubarb is a well-known herb that belongs to the Polygonaceae family, which includes approximately 60 species of the *Rheum* genus [23], including *Rheum palmatum*. *R. palmatum* has been used as a medicinal herb mainly for treating digestion problems such as constipation, vomiting, and diarrhea in Asia for over 2000 years [24,25]. Due to its pharmacological potential, *R. palmatum* is prescribed as official rhubarb in the Korean and Chinese pharmacopoeia. *R. palmatum* is endemic to China and mainly distributed in Hebei, Shanxi, Shaanxi, Gansu, Sichuan, Qinghai, and Tibet provinces, however, it has been treated as a “threatened” specie in China. Extract of *R. palmatum* has been recently reported to exert anti-inflammatory [26], antidiabetic [27], and anticancer properties [28]. According to a recent report, *R. palmatum* significantly downregulates the increased serum levels of the liver enzymes alanine aminotransferase (ALT) and aspartate aminotransferase (AST), as well as globulin and albumin levels in rats with hepatocellular carcinoma [29]. Moreover, high-fat diet-induced nonalcoholic fatty liver disease (NAFLD) is attenuated by treatment with *R. palmatum* [30]. Zhang et al. reported that *R. palmatum* exerts an effect against acute liver failure by downregulating the NF-κB signaling pathway [31]. However, the active ingredients and underlying mechanism responsible for the hepatoprotective effects of *R. palmatum* via STAT3 regulation are not fully elucidated. 

In this study, as a part of our ongoing projects to discover natural products exhibiting hepatoprotective potential [32], we found that the EtOH extract of *R. palmatum* inhibited HSC activation in our bioactive screening. Chemical investigation of the EtOH extract of *R. palmatum* led to successful isolation of chrysophanol 8-*O*-glucoside, which was determined by structural analysis using NMR spectroscopic techniques and electrospray ionization mass spectrometry (ESIMS). We confirmed that chrysophanol 8-*O*-glucoside inhibited HSC activation through analysis of α-SMA and collagen expression at the mRNA and protein levels. To elucidate the action mechanism of chrysophanol 8-*O*-glucoside on HSC activation, molecular mechanism pathways associated with HSC activation were explored using quantitative PCR (qPCR) and western blotting analysis. Herein, we report the isolation of chrysophanol 8-*O*-glucoside and its anti-fibrosis effects through regulation of the STAT3 signaling pathways.

## 2. Results

### 2.1. The Inhibitory Effects of EtOH Extract of R. palmatum on HSC Activation

**Figure 1:**
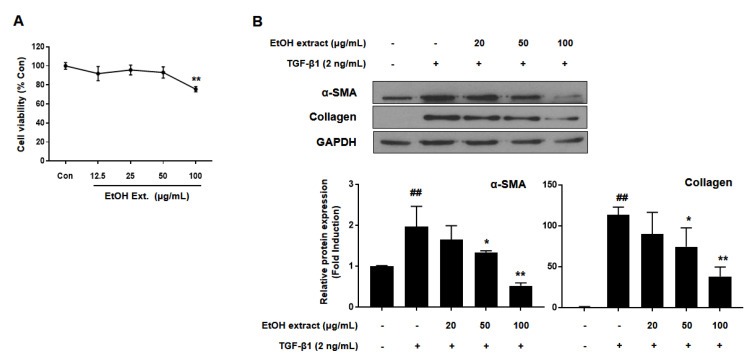
EtOH extract of *R. palmatum* inhibited the activation of LX-2 cells. (**A**) Cytotoxicity of EtOH extract of *R. palmatum* in LX-2 cells was measured by WST-1 assay. LX-2 cells were treated with EtOH extract of *R. palmatum* for 48 h after activation by TGF-β1 for 48 h. (**B**) The protein expression levels of α-smooth muscle actin (α–SMA) and collagen were analyzed by western blotting assay and quantified by Image J. Glyceraldehyde 3-phosphate dehydrogenase (GAPDH) was used as a loading control. Each experiment was repeated three times, and the values represent mean ± SD. ^##^ *p* < 0.01 compared with the control, ** *p* < 0.01, * *p* < 0.05 compared with TGF-β1-treated cells.

The rhizome of *R. palmatum* was extracted with 95% ethanol (EtOH) to obtain crude EtOH extract. First, cytotoxicity of the EtOH extract was measured by water soluble tetrazolium salts (WST-1) assay after treatment of EtOH extract for 48 h. Cytotoxicity of the EtOH extract was shown dose-dependently, which determined the appropriate concentrations (0–100 μg/mL) to test the inhibitory effects on HSC activation (Figure 1A). To identify the inhibitory effects of EtOH extract, transforming growth factor β1 (TGF-β1) was used to activate HSCs, and then the protein expression levels of α-SMA and collagen were analyzed by western blotting. As a result, the EtOH extract significantly inhibited α-SMA and collagen expression at the concentration of 50 μg/mL (Figure 1B). 

### 2.2. Chemical Identification of Chrysophanol 8-O-Glucoside

**Figure 2:**
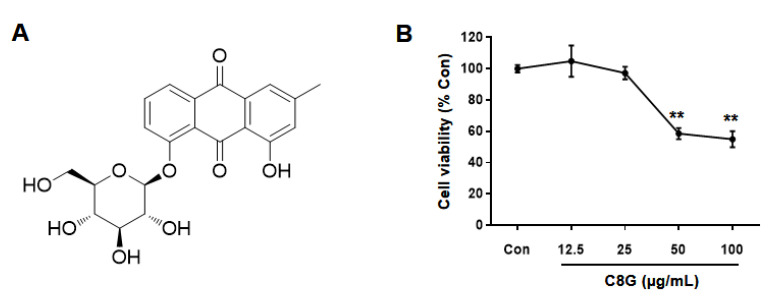
Cytotoxicity of chrysophanol 8-*O*-glucoside (C8G) in LX-2 cells. (**A**) Structure of C8G. (**B**) Cytotoxicity of C8G in LX-2 cells was assessed by WST-1 assay. LX-2 cells were treated with C8G for 48 h after activation by TGF-β1 for 48 h. Each experiment was repeated three times, and the values represent mean ± SD. ** *p* < 0.01 compared with TGF-β1-treated cells.

The EtOH extract of *R. palmatum* was divided into four different fractions: hexane-, CH_2_Cl_2_-, EtOAc-, and *n*-BuOH-soluble fractions. Liquid chromatography/mass spectrometry (LC/MS)-based analysis revealed the presence of a major peak in the EtOAc-soluble fraction [33], prompting us to conduct a chemical investigation on the EtOAc fraction. Continuous column chromatography and HPLC separation of the EtOAc fraction led to the isolation of chrysophanol 8-*O*-glucoside (C8G) (Figure 2A), which was determined by structural analysis using NMR spectroscopic techniques, including 1D (^1^H and ^13^C) NMR (Figure S1, Supplementary Materials), and LC/MS analysis (Figures S2 and S3, Supplementary Materials), and by comparing the NMR spectroscopic data with previously reported data [34].

### 2.3. Chrysophanol 8-O-Glucoside Inhibited Activation of HSCs

**Figure 3:**
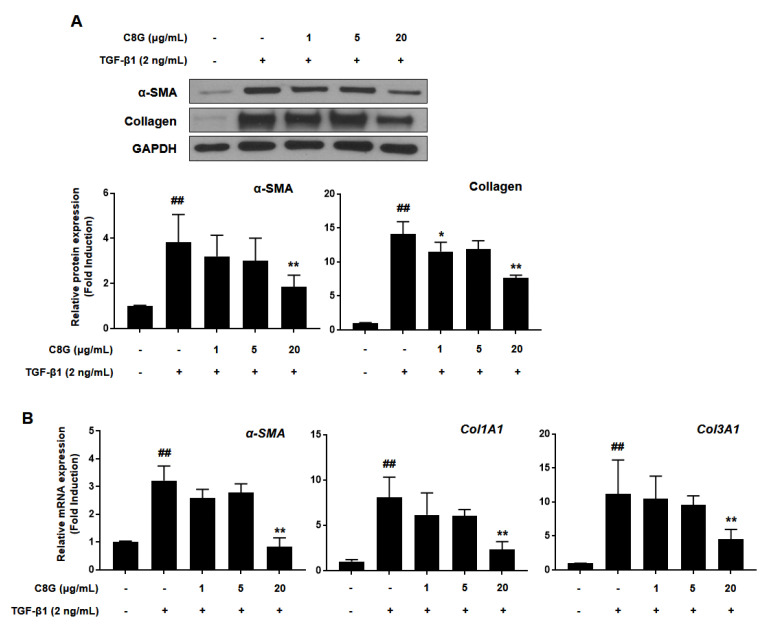
C8G suppressed the activation of LX-2 cells. Effects of C8G on LX-2 cell activation were tested by treating LX-2 cells with C8G for 48 h after activation by TGF-β1 for 48 h. (**A**) The protein expression levels of α–SMA and collagen were analyzed by western blotting assay and quantified by Image J. GAPDH was used as a loading control. (**B**) The mRNA expression levels of *α–SMA*, *Col1A1*, and *Col3A1* were measured by qPCR analysis. Each experiment was conducted under the condition of present (+) or absence (−) of C8G and TGF-β1 and repeated three times, and the values represent mean ± SD. ^##^ *p* < 0.01 compared with the control, * *p* < 0.05, ** *p* < 0.01 compared with TGF-β1-treated cells.

Before the inhibitory effects of C8G on HSC activation were investigated, cytotoxicity was tested by WST-1 assay by measuring mitochondrial dehydrogenase activity (Figure 2B). C8G at concentrations of 50 μg/mL or higher significantly decreased cell viability; therefore, C8G at 0–20 μg/mL, which did not show cytotoxicity, was used in the subsequent test. To examine the inhibitory effects of C8G on HSC activation, the protein and mRNA expression levels of α-SMA and collagen were analyzed by western blotting and qPCR analyses. The protein expression of α-SMA and collagen significantly increased after TGF-β1 treatment; however, 20 μg/mL C8G significantly suppressed this increase (Figure 3A). Similarly, 20 μg/mL C8G strongly inhibited the mRNA expression of *α-SMA*, *col1A1*, and *col3A1* induced by TGF-β1 treatment (Figure 3B).

### 2.4. Chrysophanol 8-O-Glucoside Suppressed HSC Activation through the STAT3 Signaling Pathway

**Figure 4:**
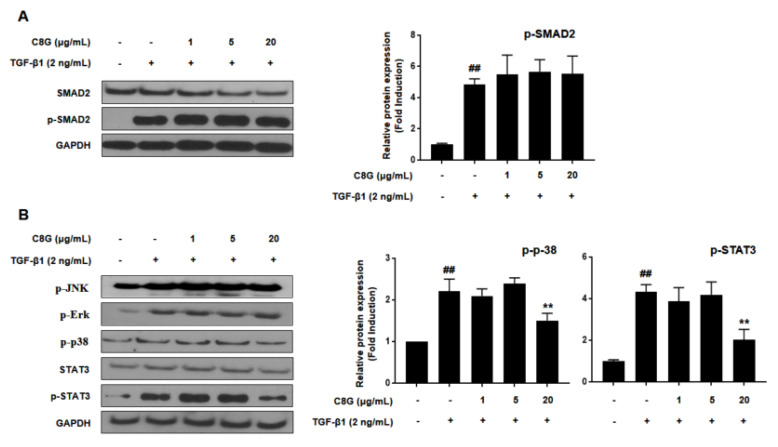
C8G suppressed STAT3 phosphorylation and translocation in LX-2 cells activated by TGF-β1. LX-2 cells were treated with C8G for 48 h after activation by TGF-β1 for 48 h. The protein expression levels of (**A**) SMAD2 and p-SMAD2 and (**B**) p-JNK, p-Erk, p-p38, STAT3, and p-STAT3 were analyzed by western blotting assay. GAPDH was used as a loading control. Each experiment was conducted under the condition of present (+) or absence (−) of C8G and TGF-β1 and repeated three times, and the values represent mean ± SD. ^##^ *p* < 0.01 compared with the control, ** *p* < 0.01 compared with TGF-β1-treated cells.

To investigate the mechanism by which C8G affected HSC activation, we analyzed the effect of C8G on the TGF-β/SMAD pathway, which is known to be associated with a canonical pathway for HSC activation by TGF-β1. TGF-β1 treatment significantly increased phosphorylated SMAD2; however, C8G did not suppress the expression of activated SMAD2 (Figure 4A). Next, we measured the levels of phosphorylated JNK (p-JNK), p-Erk, p-p38, and p-STAT3, which are proteins associated with the TGF-β non-canonical pathways of HSC activation. The results showed that the expression of p38 and p-STAT3 was significantly inhibited by C8G treatment; however, that of p-JNK and p-Erk was not reduced by C8G treatment (Figure 4B). 

**Figure 5:**
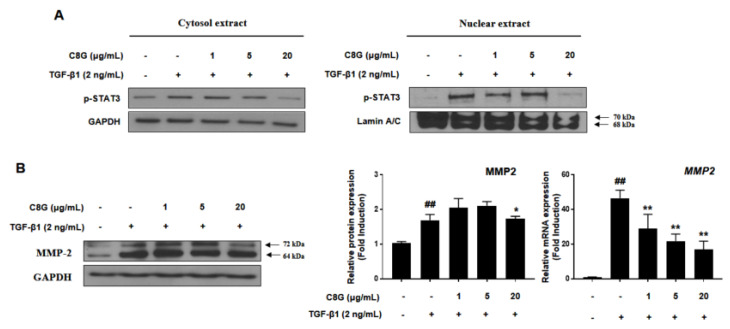
C8G inhibited nuclear translocation of p-STAT3. (**A**) The expression levels of p-STAT3 were analyzed from cytosolic and nuclear protein fraction. Lamin A/C was used as a cytosolic and nuclear loading control. (**B**) The protein and mRNA level of MMP2 was measured by qPCR and western blot analysis. Each experiment was conducted under the condition of present (+) or absence (−) of C8G and TGF-β1 and repeated three times and values represent mean ± SD. ^##^ *p* < 0.01 compared with control, * *p* < 0.05, ** *p* < 0.01, compared with TGF-β1 treatment cells.

To identify the mechanism by which C8G suppressed STAT3 signaling, the cytoplasmic and nuclear expression of p-STAT3 was measured. TGF-β1 treatment increased nuclear translocation of p-STAT3, but 20 μg/mL C8G treatment strongly inhibited p-STAT3 expression in the nucleus, indicating that C8G blocked its translocation (Figure 5A). In addition, C8G significantly inhibited the protein and mRNA expression of MMP2, which is a downstream gene of STAT3 that is important in HSC properties (Figure 5B).

## 3. Discussion

Abnormal ECM remodeling leads to ECM deposition, which is a hallmark of hepatic fibrosis [1,35]. As HSC activation after liver damage is a major source of excessive ECM deposition, inhibition of HSC activation is considered an important therapeutic strategy in hepatic fibrosis [36]. In this study, we showed that C8G strongly suppressed HSC activation through the TGF-β non-canonical pathway. C8G strongly inhibited the mRNA and protein expression of α-SMA and collagen, which are HSC activation markers, and significantly suppressed the nuclear translocation of phosphorylated STAT3. Our results suggested that C8G from *R. palmatum* can be utilized as a potential therapeutic agent for hepatic fibrosis therapy that acts by inhibiting HSC activation.

In a normal liver, HSCs are present in a quiescent state in the perisinusoidal space. Chronic damage to the liver increases the release of cytokines from hepatocytes, Kupffer cells, sinusoidal endothelial cells, and platelets, and they can activate quiescent HSCs into myofibroblast-like cells that express α-SMA, leading to ECM deposition [36]. In this study, we used TGF-β1, which is known as a cytokine that potently induces HSC activation [37,38,39]. TGF-β1 treatment transformed LX-2 cells into the activated phenotype, which highly expresses α-SMA and collagen at the mRNA and protein levels. C8G treatment significantly suppressed the expression of α-SMA and collagen and inhibited STAT3 phosphorylation. Based on the results, C8G showed a similar inhibitory potency as previously reported natural products such as artesunate [40], curcumin [41], and ginsenoside Rb1 [42]. The canonical TGF-β/SMAD signaling pathway is important in HSC activation; however, C8G did not alter the expression of SMAD2 phosphorylation, which was increased by TGF-β1 treatment [43,44]. Both mitogen-activated protein kinase (MAPK) and STAT3 signaling pathways are important in HSCs activation [45,46]. Our results showed that the expression of phosphorylated JNK, Erk, and p38 was increased by TGF-β1 treatment; however, C8G only decreased the expression of phosphorylated p38. As p38 has been reported to be a key regulator of STAT3 activation, we focused on the role of C8G on STAT3, which is associated with HSC activation [45,47]. In the recent study, chrysophanol, the aglycone of C8G, was reported to block STAT3 signaling pathways associated with cardiac hypertrophy [48]; however, to the best of our knowledge, this is the first study to show the effects of C8G on STAT3 signaling pathway. 

STAT3 plays an important role in hepatic fibrosis, and STAT3 phosphorylation was observed in the liver samples of patients with liver fibrosis and cirrhosis [49,50]. STAT3 can be activated by TGF-β1 treatment both in a SMAD-dependent and -independent manner, and it is known as a key therapeutic target in hepatic fibrosis [18,51,52,53]. TGF-β1 phosphorylates STAT3, forms dimer with STAT1 or itself, translocates to the nucleus, and then induces the transcription of downstream genes [54]. Our results indicated that C8G significantly suppressed the translocation of phosphorylated STAT3 and the expression of its downstream gene, MMP2. MMP2 is reported as an important gene in the activation and invasiveness of HSCs as well as in the accumulation of ECM [55,56]. The blocking of MMP2 expression by C8G implies that C8G can inhibit activating signals from hepatocyte when liver cells are damaged [57].

Previously, rhubarb was reported to exert either a hepatotoxic or hepatoprotective effect depending on its dose [58]. Administration of rhubarb extract has been reported to induce hepatotoxicity in rats [59,60]. On the contrary, crude rhubarb extract has been reported to alleviate alcohol-induced steatohepatitis by reducing liver inflammation and hepatic triglyceride content [61]. In addition, *R. rhabarbarum*, another plant used as rhubarb, showed protective effects on cholestatic hepatitis and hepatic fibrosis by protecting hepatocytes and decreasing oxidative stress, respectively [62,63]. However, the reason for this dual effect of rhubarb on the liver is not fully understood. Recently, Lin et al. [51] reported that C8G from a rhubarb extract induced cytotoxicity in hepatocytes by increasing reactive oxygen species (ROS) and mitochondrial damage. Chronic damage such as that caused by the hepatitis C virus or alcohol lead to the death of hepatocytes and the secretion of cytokines that can activate HSCs and hepatic fibrosis [56,64]. However, our results revealed that C8G strongly inhibited HSC activation by suppressing the STAT3 pathway, suggesting that C8G exerted protective effects against chronic liver disease.

## 4. Materials and Methods

### 4.1. Extraction and Isolation

The rhizome of *R. palmatum* (500 g) was partially chopped and extracted with 95% EtOH for 2 days twice at room temperature. The extracts were then filtered, and the filtrate was concentrated under vacuum pressure, generating a crude EtOH extract (37.5 g). The crude extract was dissolved in distilled water (800 mL) and further subjected to solvent partition with hexane, CH_2_Cl_2_, EtOAc, and *n*-BuOH, which yielded solvent-soluble fractions. LC/MS-guided analysis of the fractions revealed the presence of a major peak in the EtOAc-soluble fraction [33,65]. The EtOAc-soluble fraction (11.6 g) was fractionated on silica gel column chromatography with a gradient solvent system from hexane-EtOAc (10:1 to 1:1, *v*/*v*) to CH_2_Cl_2_-MeOH (1:1, *v*/*v*) to obtain seven sub-fractions (E1–E7). Sub-fraction E3 (1.2 g) was separated in a silica gel column chromatography using a gradient solvent system from CH_2_Cl_2_-MeOH (20:1 to 1:1, *v*/*v*) to 100% MeOH to obtain five sub-fractions (E31–E35). Sub-fraction E31 (200.4 mg) was separated by preparative HPLC using Hector-C18 column with a gradient solvent system from MeOH-H_2_O (1:1 to 1:0, *v*/*v*; flow rate: 5 mL/min) to yield five sub-fractions (E311–E315). Chrysophanol 8-*O*-glucoside (10.6 mg; *t_R_* = 27.0 min, 0.028%) was obtained from sub-fraction E313 (61.4 mg) by semi-preparative HPLC with a Phenomenex Luna phenyl-hexyl column (67% MeOH; flow rate: 2 mL/min).

### 4.2. Cell Viability Analysis

Cell viability was analyzed by a WST-1 assay (Roche, Mannheim, Germany) [66,67]. LX-2 cells were seeded onto 96-well plates at a density of 1 × 10^4^ cells/well and incubated for 24 h. Cells were treated with the study substance for 48 h following TGF-β1 induction. A WST-1 reagent was added to each well according to the manufacturer’s instructions and incubated within 30 min at 37 °C. Absorbance was measured at 440 nm and 690 nm using a microplate reader (Molecular Devices, Sunnyvale, CA, USA).

### 4.3. Comparative qPCR

Total RNA was isolated using TRIzol reagent (Life Technologies, Grand Island, NY, USA) following the protocol provided by the manufacturer [68,69]. RNA concentration was measured using BioDrop Duo (BioDrop, Cambridge, UK), and cDNA was synthesized by using a High-Capacity cDNA Reverse Transcription System (Life Technologies). qPCR was performed in duplicate for each sample using SYBR^®^ Premix Ex Taq^TM^ (Life Technologies) and a CFX CFX96 Real-Time PCR System (Bio-Rad, Hercules, CA, USA). qPCR was performed using the primers listed in Table 1. The mRNA expression level of genes of interest was normalized to that of GAPDH. 

### 4.4. Western Blotting Analysis

LX-2 cells were seeded in a six-well plate at 5 × 10^4^ cells/well and incubated for 24 h. After pretreatment with TGF-β1 for 48 h, the cells were treated with chrysophanol 8-*O*-glucoside for 48 h, washed twice with phosphate-buffered saline (PBS), and lysed with radioimmunoprecipitation assay buffer (Thermo Scientific, Waltham, MA, USA) with a protease inhibitor cocktail (GenDEPOT, Barker, TX, USA), phosphate inhibitor (BioVision, Milpitas, CA, USA), and 0.1% sodium dodecyl sulphate (SDS). Cell lysates were incubated on ice for 30 min and subsequently centrifuged at 13,000× *g* for 15 min at 4 °C. The Pierce^TM^ BCA Protein Assay Kit (Pierce, Rockford, IL, USA) was used to quantify protein concentration in the cell lysates. Samples were denatured with buffer containing 2% sodium dodecyl sulphate (SDS), 6% 2-mercaptoethanol, 40% glycerol, 0.004% bromophenol blue, and 0.06 M Tris–HCl at 90–100 °C for 6 min, then cooled at 20–25 °C for 5 min. A 15-μg sample of each protein was resolved in 10% or 8–16% gradient SDS-polyacrylamide gel electrophoresis (PAGE) gel (Bio-Rad) and transferred to a polyvinylidene fluoride (PVDF) membrane (Bio-Rad). The membrane was blocked with 5% skim milk in tris-buffered saline with Tween 20 (TBS-T) at 20–25 °C for 1 h and incubated with primary antibodies overnight at 4 °C. Next, the membranes were washed with TBS-T and incubated for 1 h with secondary antibodies conjugated to horseradish peroxidase. Protein bands were then developed with enhanced chemiluminescence reagents (Bio-Rad) using an automatic X-ray film processor (JPI Healthcare, Seoul, Korea). The densities of each band were normalized to those of a GAPDH band: Anti-collagen (ab138492; Abcam, Cambridge, MA, USA), anti-MMP2 (ab37150; Abcam), anti-alpha-SMA (ab5694; Abcam), anti-STAT3 (#9139; Cell Signaling, San Jose, CA, USA), anti-p-STAT3 (#9145; Cell Signaling), anti-SMAD2 (#5339; Cell Signaling), anti-p-SMAD2 (#3108; Cell Signaling), p-JNK (#9251S; Cell signaling), p-Erk (#4370; Cell Signaling), p-p38 (#9211; Cell Signaling), and anti-GAPDH (015-25473; Wako Pure Chemical Industries, Osaka, Japan) were used in the western blotting analysis.

### 4.5. Preparation of Nuclear Extracts

LX-2 cells were washed with ice-cold PBS and then lysed with hypotonic buffer (10 mM HEPES (pH 7.9), 10 mM KCl, 1.5 mM MgCl_2_, 1 mM EDTA, 10 mM protease inhibitor cocktail, 10 mM protein phosphatase inhibitors) with 0.75% NP-40 on ice for 15 min. After centrifugation at 3000 rpm for 5 min at 4 °C, the obtained cell pellets were rinsed with hypotonic buffer and then resuspended in high-salt buffer (20 mM HEPES (pH 7.9), 0.4 M NaCl, 1 mM EDTA, glycerol 25%) at 4 °C for 15 min. Nuclear extracts were collected from the supernatants by centrifugation at 13,000× *g* for 5 min at 4 °C.

## 5. Conclusions

Our study showed, for the first time, the inhibitory effects of C8G from *R. palmatum* on HSC activation through inhibition of the STAT3 pathway, which is a key therapeutic target in hepatic fibrosis, thus providing experimental evidence for the hepatoprotective effects of C8G against liver damage. Collectively, these data suggest that C8G can be an active compound responsible for the hepatoprotective effects of *R. palmatum* and provide insights into the usage of *R. palmatum* for liver disease.

## Figures and Tables

**Table 1:** Lists of qPCR primers.

Gene	Forward	Reverse
α-SMA	CTGGCATCGTGCTGGACTCT	GATCTCGGCCAGCCAGATC
MMP2	GAGAACCAAAGTCTGAAGAG	GGAGTGAGAATGCTGATTAG
Collagen 1A1	GGCAACAGCCGCTTCACCTAC	GCGGGAGGACTTGGTGGTTTT
Collagen 3A1	CACGGAAACACTGGTGGACAGATT	ATGCCAGCTGCACATCAAGGAC
GAPDH	AATCCCATCACCATCTTCCA	TGGACTCCACGACGTACTCA

## Supplementary Materials

The following are available online at https://www.mdpi.com/1422-0067/21/23/9044/s1: Materials and Methods, including general experimental procedures, sample material, cell culture, and statistical analysis. Figure S1: The ^1^H NMR spectrum of chrysophanol 8-*O*-glucoside (CD_3_OD, 700 MHz); Figure S2: LC/MS data of chrysophanol 8-*O*-glucoside; Figure S3: (A) UV chromatogram of LC/MS (detection wavelength was set as 254 nm) of chrysophanol 8-*O*-glucoside. (B) UV chromatogram of LC/MS (detection wavelength was set as 254 nm) of crude EtOH extract.
