# Supplementary material for: Hepatoprotective Potency of Chrysophanol 8-O-Glucoside from Rheum palmatum L. against Hepatic Fibrosis via Regulation of the STAT3 Signaling Pathway
Source: Int J Mol Sci. 2020 Nov 27;21(23):9044. doi: 10.3390/ijms21239044 (PMC7730872; doi:10.3390/ijms21239044)
Supplement: Supplementary file 1 [file ijms-21-09044-s001.pdf]

Supplementary Material

Hepatoprotective Potency of Chrysophanol 8-*O*-Glucoside from *Rheum palmatum* L. against Hepatic Fibrosis via Regulation of the STAT3 Signaling Pathway

Yong Joo Park [1,a], Kwang Ho Lee [1,a], Mi Seon Jeon [1], Yong Hoon Lee [1], Yoon-Joo Ko [2], Changhyun Pang [3], Bonglee Kim [4], Kyu Hyuck Chung [1,*], and Ki Hyun Kim [1,*]

[1] School of Pharmacy, Sungkyunkwan University, Suwon 16419, Republic of Korea; pyj084@msn.com (Y.J.P); sholaly@naver.com (K.H.L.); jmsun123@naver.com (M.S.J.); yonghoon2090@gmail.com (Y.H.L.)
[2] Laboratory of Nuclear Magnetic Resonance, National Center for Inter-University Research Facilities (NCIRF), Seoul National University, Gwanak-gu, Seoul 08826, Republic of Korea; yjko@snu.ac.kr (Y.J.K.)
[3] School of Chemical Engineering, Sungkyunkwan University, Suwon 16419, Republic of Korea; chpang@skku.edu (C.P.)
[4] Department of Pathology, College of Korean Medicine, Kyung Hee University, Seoul 02453, Republic of Korea; bongleekim@khu.ac.kr (B.K.)

* Correspondence: khchung@skku.edu (K.H.C.); Tel.: +82-31-290-7714; khkim83@skku.edu (K.H.K.); Tel.: +82-31-290-7700

General experimental procedures

Ultraviolet (UV) spectra were determined using an Agilent 8453 UV-visible spectrophotometer (Agilent Technologies, Santa Clara, CA, USA). Infrared (IR) spectra were acquired using a Bruker IFS−66/S FT-IR spectrometer, and NMR spectra were measured using a Bruker Avance III HD 800 NMR spectrometer with a 5 mm TCI Cryoprobe (Bruker, Karlsruhe, Germany). Preparative high-performance liquid chromatography (HPLC) and semi-preparative HPLC (Waters Corporation, Milford, CT, USA) were performed using a Hector-C18 column (250 × 21.2 mm, 7 μm) and a Phenomenex Luna Phenyl−hexyl 100 A column (250 × 10 mm, 10 μm), respectively, with a Waters 1525 binary HPLC pump equipped with a Waters 996 photodiode array detector. LC/MS analysis was performed on an Agilent 1200 Series HPLC system (Agilent Technologies) equipped with a diode array detector and a 6130 Series ESI mass spectrometer using an analytical Kinetex C18 100 Å column (100 × 2.1 mm i.d., 5 µm; Phenomenex, Torrance, CA, USA). Column chromatography was performed with a silica gel 60 (230–400 mesh; Merck) and an RP-C18 silica gel column (230–400 mesh; Merck). First-grade solvents (Samchun Pure Chemicals Co., Ltd., Pyeongtaek, Korea) were used for fractionation and isolation. Merck precoated silica gel F_{254} plates and reverse-phase (RP)-18 F_{254s} plates were used for thin-layer chromatography (TLC). Spots were detected on TLC under UV light (dual wavelength 254/365 nm) or by heating after spraying with anisaldehyde-sulfuric acid.

Sample material

R. palmatum was purchased at Kyungdong herbal market, Korea (Seoul), in June 2019. The samples were identified by one of the authors (Prof. K. H. Kim). A voucher specimen (SKKU DW-2019-06) has been stored in the herbarium of the School of Pharmacy, Sungkyunkwan University, Korea (Suwon).

Cell culture

LX-2 human HSCs were obtained from the American Type Culture Collection (Manassas, VA, USA). Cells were cultured in Dulbecco's modified Eagle medium (Sigma, St. Louis, MO, USA) supplemented with 10 % of fetal bovine serum (Biotechnics Research Inc., Lake Forest, CA, USA), penicillin (100 units/mL), and streptomycin (100 µg/mL). Cells were maintained at 37°C in an atmosphere containing 95 % air and saturation humidity of 5 % CO_2.

Statistical analysis

Data were analyzed using GraphPad Prism version 7.00 (GraphPad software Inc., San Diego, CA, USA) and Excel (Microsoft, Redmond, WA, USA). Each assay was performed a minimum of three times. Data from each assay were expressed as mean ± standard deviation (SD). Differences between the groups were assessed by Duncan's *post-hoc* test after one-way analysis of variance. Statistical significance was accepted at $p < 0.05$.

Figure S1. The ^{1}H NMR spectrum of chrysophanol 8-*O*-glucoside (CD_3OD, 700 MHz)

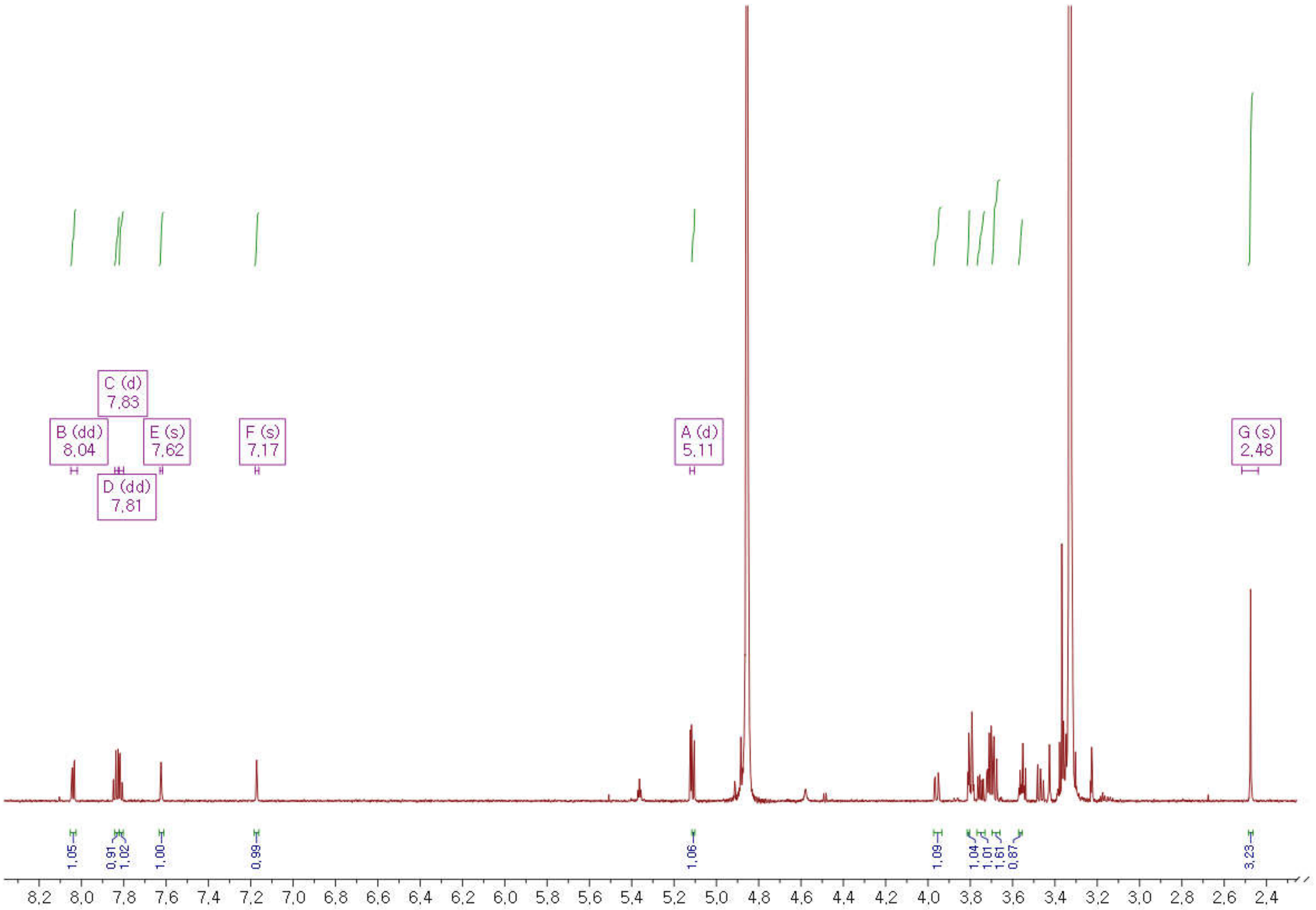

Chrysophanol 8-O-glucoside

Yellowish powder. ^{1}H NMR (CD_3OD, 700 MHz): δ 8.04 (1H, dd, *J* = 7.5, 1.5 Hz, H-5), 7.83 (1H, dd, *J* = 8.5, 7.5 Hz, H-6), 7.81 (1H, dd, *J* = 8.5, 1.5 Hz, H-7), 7.62 (1H, br s, H-4), 7.17 (1H, br s, H-2), 5.11 (1H, d, *J* = 7.5 Hz, anomeric-H), 3.96–3.56 (sugar-H), 2.48 (3H, s, Ar-CH_3); ESIMS (negative-ion mode) *m/z* = 415.0 $[M – H]^-$.

Figure S2. LC/MS data of chrysophanol 8-*O*-glucoside

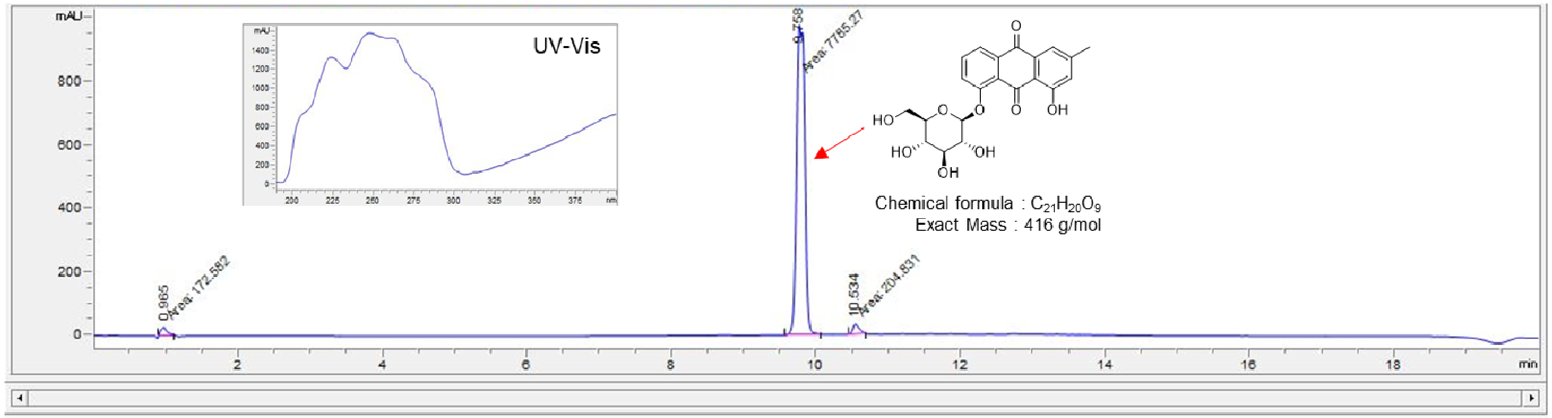

#	Time	Area	Height	Width	Area%	Symmetry
1	0.965	172.6	26.5	0.1084	2.114	0.703
2	9.758	7785.3	977.9	0.1327	95.376	0.55
3	10.534	204.8	32.6	0.1046	2.509	0.602

Purity : 95.376%

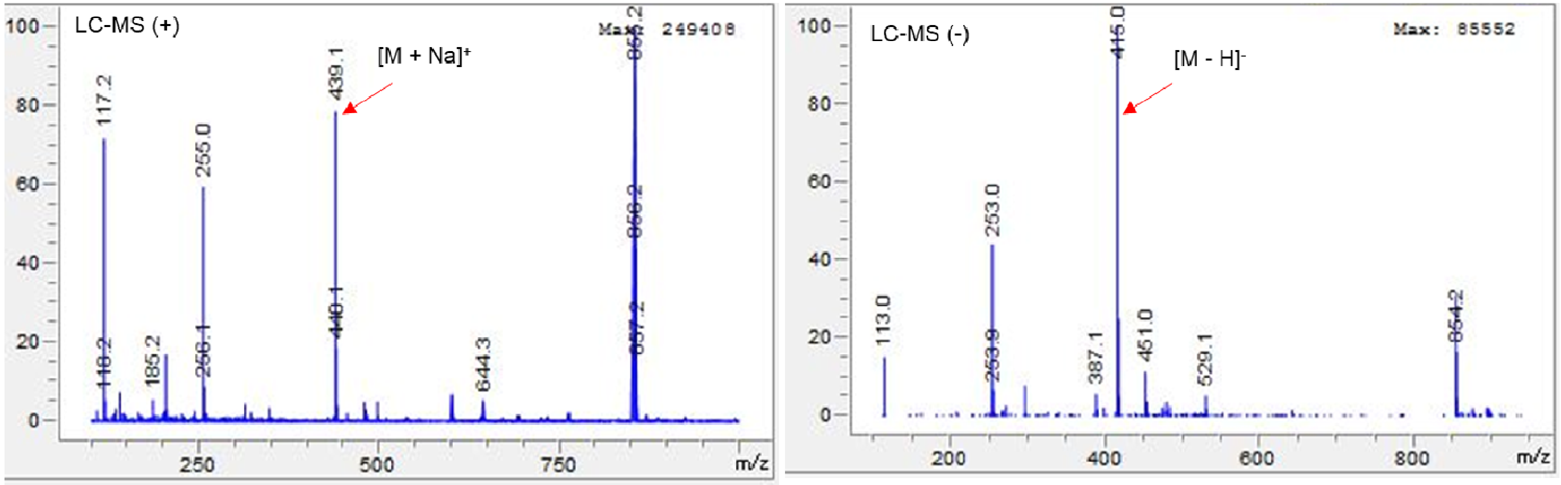

LC/MS analysis

LC/MS analysis for chrysophanol 8-*O*-glucoside was performed by LC/MS (Agilent Technologies, Santa Clara, CA, USA) using a LC-MS Agilent 1200 Series analytical system equipped with a photodiode array (PDA) detector combined with a 6130 Series ESI mass spectrometer. Analysis was performed by injection of 10 μL of chrysophanol 8-*O*-glucoside using a Kinetex C18 column (2.1 × 100 mm, 5 μm; Phenomenex, Torrance, CA, USA) set at 25°C. The mobile phase consisting of formic acid in H_2O [0.1% (v/v)] (A) and methanol (B) was delivered at a flow rate of 0.3 mL/min by applying the following programmed gradient elution: 10%-100% (B) for 10 min, 100% (B) isocratic for 5 min, and then 100% to 10% (B) for 5 min, to perform post-run reconditioning of the column.

Figure S3. (A) UV chromatogram of LC/MS (detection wavelength was set as 254 nm) of chrysophanol 8-*O*-glucoside. (B) UV chromatogram of LC/MS (detection wavelength was set as 254 nm) of crude EtOH extract.

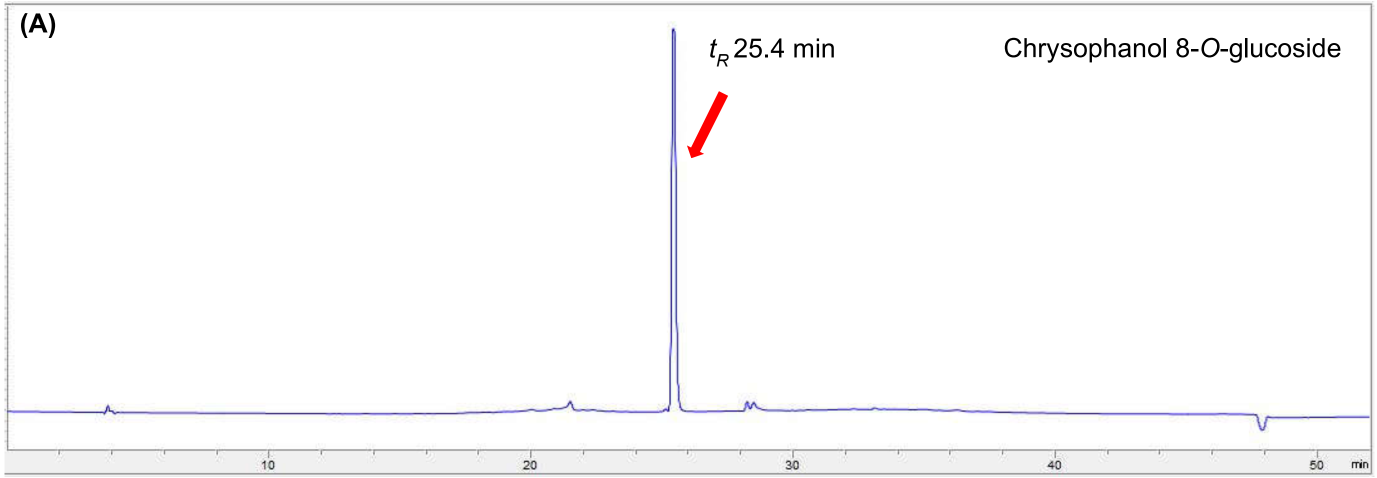

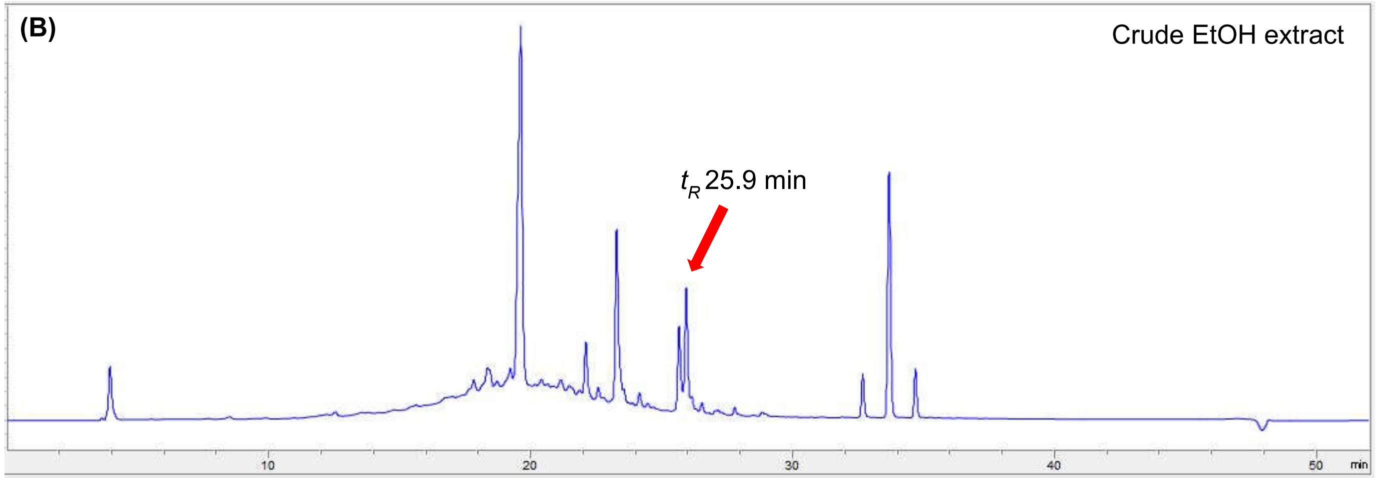

LC/MS analysis

LC/MS analysis for crude EtOH extract and chrysophanol 8-*O*-glucoside was performed by the same LC/MS equipment (Agilent Technologies, Santa Clara, CA, USA). Analysis was performed by injection of 10 μL of samples using Agilent Eclipse Plus C18 column (4.6 × 100 mm, 3.5 μm; Agilent Technologies) set at 25°C. The mobile phase consisting of formic acid in H_2O [0.1% (v/v)] (A) and methanol (B) was delivered at a flow rate of 0.3 mL/min by applying the following programmed gradient elution: 0%-100% (B) for 30 min, 100% (B) isocratic for 10 min, 10% (B) for 1 min, and then 10% (B) isocratic for 9 min, to perform post-run reconditioning of the column.
